# Ex Vivo Mitochondrial Respiration Parallels Biochemical Response to Ibrutinib in CLL Cells

**DOI:** 10.3390/cancers13020354

**Published:** 2021-01-19

**Authors:** Subir Roy Chowdhury, Cheryl Peltier, Sen Hou, Amandeep Singh, James B. Johnston, Spencer B. Gibson, Aaron J. Marshall, Versha Banerji

**Affiliations:** 1 Research Institute in Oncology and Hematology, CancerCare Manitoba, Winnipeg, MB R3V 0V9, Canada; sroychowdhury@cancercare.mb.ca (S.R.C.); Cheryl.Peltier@umanitoba.ca (C.P.); singha36@myumanitoba.ca (A.S.); jjohnsto@cancercare.mb.ca (J.B.J.); spencer.gibson@umanitoba.ca (S.B.G.); Aaron.Marshall@umanitoba.ca (A.J.M.); 2 Department of Immunology, Max Rady College of Medicine, Rady Faculty of Health Sciences, University of Manitoba, Winnipeg, MB R3E 0T5, Canada; Sen.Hou@umanitoba.ca; 3 Biochemistry and Medical Genetics, Max Rady College of Medicine, Rady Faculty of Health Sciences, University of Manitoba, Winnipeg, MB R3E 3N4, Canada; 4 Internal Medicine, Max Rady College of Medicine, Rady Faculty of Health Sciences, University of Manitoba, Winnipeg, MB R3E 3P4, Canada

**Keywords:** mitochondrial respiration, chronic lymphocytic leukemia (CLL), B-cell receptor (BCR), ibrutinib (IB), Bruton tyrosine kinase (BTK) inhibitor, plasma chemokine (C-C motif) ligands 3 and 4 (CCL3 and CCL4), β-2 microglobulin (β-2 M), lactate dehydrogenase (LDH)

## Abstract

**Simple Summary:**

Chronic lymphocytic leukemia (CLL) is a cancer that is characterized by dysfunctional mitochondria. This results in a deranged mitochondrial metabolism. Many drugs are tested using simple live or dead cell assays and as such the impact on the altered mitochondrial function is not evaluated. One such drug is ibrutinib. The use of ibrutinib at full doses can lead to significant side effects resulting in dose reduction or even discontinuation of the drug in clinical practice. In this study, we reviewed the effect of the dose of ibrutinib on mitochondrial function of the CLL cells from patients treated with ibrutinib and did not observe any difference in the mitochondrial respiration. We also evaluated the effect of progression of CLL cells from patients on ibrutinib treatment and the impact on mitochondrial respiration. We believe our findings are novel and suggest that evaluation of mitochondrial function of CLL cells is of importance to help design safe treatments in the preclinical setting.

**Abstract:**

Mitochondrial respiration is becoming more commonly used as a preclinical tool and potential biomarker for chronic lymphocytic leukemia (CLL) and activated B-cell receptor (BCR) signaling. However, respiration parameters have not been evaluated with respect to dose of ibrutinib given in clinical practice or the effect of progression on ibrutinib treatment on respiration of CLL cells. We evaluated the impact of low and standard dose ibrutinib on CLL cells from patients treated in vivo on mitochondrial respiration using Oroboros oxygraph. Cytokines CCL3 and CCL4 were evaluated using the Mesoscale. Western blot analysis was used to evaluate the BCR and apoptotic pathways. We observed no difference in the mitochondrial respiration rates or levels of plasma chemokine (C-C motif) ligands 3 and 4 (CCL3/CCL4), β-2 microglobulin (β-2 M) and lactate dehydrogenase (LDH) between low and standard doses of ibrutinib. This may confirm why clinical observations of the safety and efficacy of low dose ibrutinib are observed in practice. Of interest, we also observed that the mitochondrial respiration of CLL cells paralleled the increase in β-2 M and LDH at progression. Our study further supports mitochondrial respiration as a biomarker for response and progression on ibrutinib in CLL cells and a valuable pre-clinical tool.

## 1. Introduction

Ibrutinib, a Bruton tyrosine kinase (BTK) inhibitor, has become a standard in the treatment of chronic lymphocytic leukemia (CLL) patients [1,2]. There are ongoing debates as to the best dose. Bose et al. demonstrated that BTK occupancy occurs at lower doses of ibrutinib of ~170 mg OD (standard dosing is 420 mg per day). Chen at el. conducted a pilot study of 12 patients where a stepwise dose reduction of ibrutinib did not alter pharmacokinetics (PK), nor BTK occupancy, significantly [3,4,5]. In addition, phosphorylated BTK (pBTK) and total BTK (tBTK) protein levels were decreased at both standard and reduced doses of ibrutinib (420 vs. 140, 280 mg per day) [4]. In our retrospective clinical real world evidence study, we followed a cohort of 64 patients where approximately 60% of ibrutinib treated patients were either dose reduced or on a lower dose of ibrutinib, mainly due to toxicities or physician preferences [6]. The discontinuation rates in this study by Uminski et al. [6] were lower than published in clinical trials with similar results recently reported by the Mayo Clinic [7,8], indicating that ibrutinib dose reductions related to toxicities are common in real world clinical experiences.

In a translational study, we recently demonstrated that mitochondrial respiration is a biomarker for CLL and active B-cell receptor (BCR) signaling in a cohort of 81 CLL patients and decreased with ibrutinib treatment in a small cohort of treated patients [9]. Given the observations with the dose reduction in our real world cohort and parallel reports of dose reductions, we went back and reviewed the effect of ibrutinib dose on mitochondrial respiration parameters at both standard and reduced doses for this study. We evaluated the impact of ibrutinib dose on BTK protein and its phosphorylation levels, as well as levels of chemokines CCL3 and CCL4 which are known biomarkers for BCR signaling and drug response [10,11]. Correlation with clinical parameters such as β-2 M and lactate dehydrogenase (LDH), which have been reported as biomarkers of disease progression and response to ibrutinib therapy, were also evaluated in parallel [12]. Furthermore, observed increases in CLL cell mitochondrial respiration in parallel with biochemical markers from patients who relapsed while on ibrutinib therapy confirms mitochondrial respiration as a biomarker of disease activity.

## 2. Results

**Figure 1:**
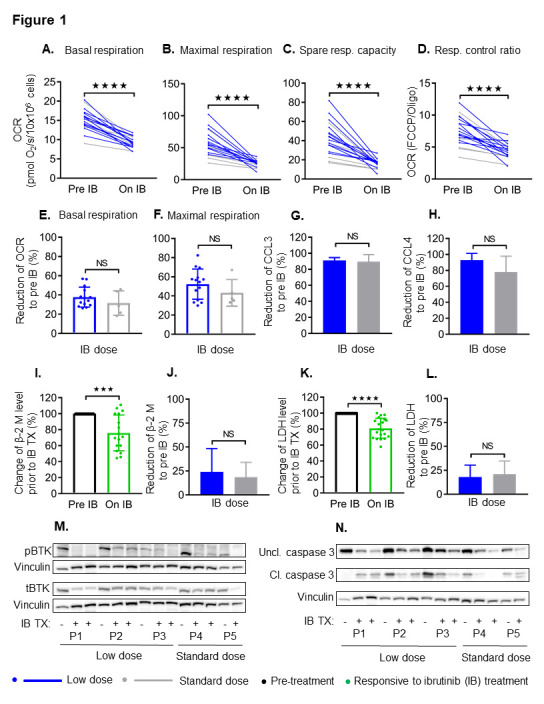
Ibrutinib has similar effects on mitochondrial respiration profiles, CCL3 and 4, β-2 M, lactate dehydrogenase (LDH), Bruton tyrosine kinase (BTK) signaling and caspase-3 cleavage in chronic lymphocytic leukemia (CLL) patients independent of dose. The effect of ibrutinib treatment on basal respiration (**A**), maximal respiration (**B**), spare respiratory capacity (**C**), and respiratory control ratio (**D**) in primary CLL cells from patients pre-treatment and on ibrutinib treatment with low (blue) or standard (grey) dose. N = 14 for low dose and N = 5 for standard dose of ibrutinib. Values are mean ± S.D. **** *p* < 0.0001 (paired two-tailed Student’s *t*-test). The reduction of mitochondrial respiration parameters with low and standard dose of ibrutinib treatment is summarized for basal respiration (**E**) and maximal respiration (**F**) in freshly isolated CLL cells. N = 14 for low dose and N = 5 for standard dose of ibrutinib. Values are mean ± S.D. *** *p* < 0.0005, NS: non-significant (unpaired two-tailed Student’s *t*-test). In parallel, there is a reduction of chemokine ligands CCL3 (**G**) and CCL4 (**H**) in ibrutinib treated CLL patient plasma samples by dose. Decreased levels of β-2 M and LDH in ibrutinib treated patients compared to pre-treatment (**I**,**K**) and the decrease of β-2 M or LDH was similar to low or standard dose ibrutinib CLL patients (**J**,**L**). Demonstration of effects on pBTK, tBTK (**M**), and caspase-3 cleavage (**N**) are independent of dose. OCR: Oxygen consumption rate; resp: Respiratory; IB: Ibrutinib; TX: Treatment; Uncl. caspase-3: Uncleaved caspase-3; Cl. caspase-3: Cleaved caspase-3.

We retrospectively reviewed paired pre-treatment and on-treatment ibrutinib samples to determine the effect of ibrutinib dose on mitochondrial respiration rates and CCL3 and CCL4 (Figure 1A–H). All mitochondrial respiration parameters—basal respiration, mitochondrial respiration, spare respiratory capacity, and respiratory control ratio, were reduced by ibrutinib treatment, regardless of dose (Figure 1A–D). When evaluating the trajectories of respiration parameters across various time points, there are no differences in kinetics between the low and standard ibrutinib dose (Supplementary Figure S1A,B). The magnitude of reduction in mitochondrial respiration parameters was also not significantly different between low and standard dose treatment groups (Figure 1E,F and Supplementary Figure S1C,D).

To support the mitochondrial respiration data, levels of chemokines CCL3 and CCL4 as a surrogate for BCR signaling were not impacted by dose (Figure 1G,H). In addition, we evaluated the levels of β-2 M and LDH, which are significantly decreased in ibrutinib treated patients (Figure 1I,K) and reduced to a similar extent despite dose (Figure 1J,L). The levels of CCL3 (91.14 ± 1.71 vs. 89.83 ± 4.30), CCL4 (92.97 ± 4.23 vs. 78.09 ± 9.89), β-2 M (23.96 ± 6.53 vs. 18.45 ± 7.79), and LDH (18.09 ± 3.29 vs. 21.06 ± 5.59) were similarly decreased with low or standard dose of ibrutinib compared to pre-treatment levels. When evaluating the target of ibrutinib, we observed consistent reduction in phosphorylated BTK (pBTK at Tyr223), total BTK (tBTK) protein, and their ratio (Figure 1M). Reduction in pBTK and tBTK levels was similar despite the low or standard dose of ibrutinib (Supplementary Figure S1E,F). Apoptosis occurred similarly across both groups as demonstrated by caspase 3 cleavage (Figure 1N). These data demonstrate the comparable activity of the drug on the target despite reduced dosing.

**Figure 2:**
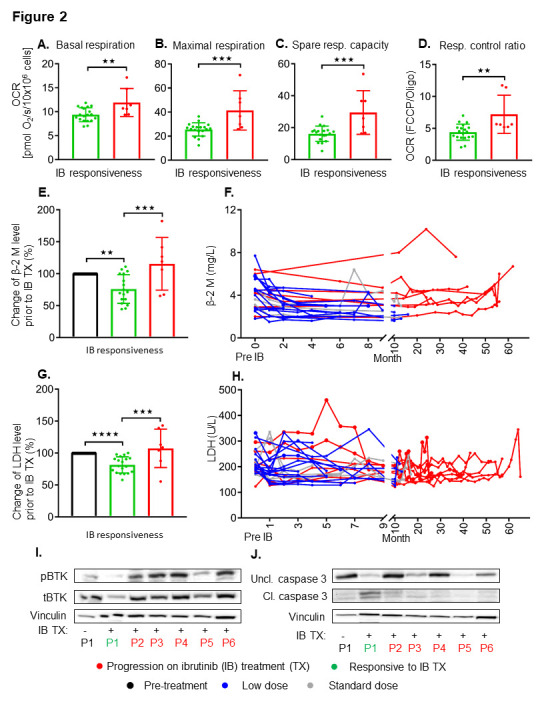
Patients progressed while on ibrutinib treatment have increased mitochondrial respiration profiles, β-2 M, LDH, and loss of BCR signaling suppression and caspase-3 cleavage. Basal respiration (**A**), maximal respiration (**B**), spare respiratory capacity (**C**), and respiratory control ratio (**D**) are summarized in freshly isolated CLL cells from ibrutinib-sensitive (green) and patients who have progressed on ibrutinib (red). Values are mean ± S.D., ibrutinib- sensitive, N = 19, and ibrutinib-progressed, N = 7. ** *p* < 0.005, *** *p* < 0.0005 (unpaired two-tailed Student’s *t*-test). Plasma levels of β-2 M (**E**) and LDH (**G**) are summarized from pre-treatment (black), ibrutinib-sensitive (green) and progressed on ibrutinib treated patients (red). Values are mean ± S.D., ibrutinib-sensitive, N = 19, and ibrutinib-progressed, N = 7, pre-treatment, N = 26. ** *p* < 0.005, *** *p* < 0.0005, **** *p* < 0.0001 (one way ANOVA with Tukey’s post hoc test). Time-dependent effects of ibrutinib treated samples are shown on β-2 M (**F**) and LDH (**H**) level with low (blue), standard (grey) dose, and with progression on ibrutinib treatment (red). Western blot analysis demonstrating the effect of ibrutinib on pBTK, tBTK (**I**) and caspase-3 cleavage (**J**) in progressed samples (**H**). Protein levels of pBTK/tBTK ratios and cleaved/uncleaved caspase-3 ratios in ibrutinib-sensitive (P1 in green) and ibrutinib-progressed (P2-P6 in red) compared to pre-treatment sample (P1 in black).

The technology for mitochondrial respiration is novel to the field of CLL and was not available when patients who are currently progressing on ibrutinib therapy first initiated treatment, thus we were unable to match the samples with a pre-treatment sample as it requires fresh CLL cells. This being said, CLL cells from patients who had progressed on ibrutinib therapy were evaluated at the time of relapse while on therapy (i.e., drug was not stopped) (duration 26–62 months on treatment at both standard and low doses) and were found to have increased mitochondrial respiration rates across all parameters (Figure 2A–D), and increased β-2 M and LDH levels at the time of progression (Figure 2E,G). Given, that the β-2 M correlated with response on therapy, we evaluated the trajectory by ibrutinib responsive or progressed samples and superimposed them. β-2 M and LDH is highlighted in Figure 2F,H for responders along with progressors, respectively. We also demonstrate an increase in pBTK and tBTK (Figure 2I and Supplementary Figure S2A) and loss of caspase 3 cleavage (Figure 2J and Supplementary Figure S2B) in samples which have progressed on ibrutinib treatment compared to P1, an ibrutinib responsive sample.

## 3. Discussion

Significant literature on dose reductions has emerged due to the toxicities of ibrutinib in CLL, in addition to a stepwise dose reduction phase 2 clinical trial [6,7,8]. Our data suggest that mitochondrial bioenergetics of CLL cells, as well as their production of chemokines, and plasma β-2 M and LDH, are similarly altered regardless of dose of ibrutinib in responding patients [5,12]. Furthermore, CLL progression on ibrutinib therapy results in increases in the mitochondrial respiration profile and correlates with an increase in β-2 M and LDH regardless of duration on therapy or dose of ibrutinib at time of progression [12]. This blinded analysis of ibrutinib dose further supports the rationale for dose reductions based on the use of a novel biomarker and mitochondrial respiration [3,4,5,6,8,9,13].

With ibrutinib’s increasing prevalence in clinical practice due to its superiority in head to head comparison to chemo-immunotherapy and increased clinical uptake, dose considerations are even more important as it applies to the majority of CLL patients that are older and often have other comorbid conditions and thus potential increased risk for toxicity. At this time there is much need for a formal evaluation of dose reductions to determine if toxicities are diminished and duration of remission is similar. Secondly, how these compare to second generation BTK inhibitors, such as acalabrutinib, and their toxicity profile and efficacy is of great interest. A head to head study evaluating 1st generation vs. 2nd generation BTK inhibitors will be reported in the near future. However, for now, we believe, based on our preclinical data and the many real word evidence based reports, that dose reductions are safe in certain populations.

With respect to progression, as we are now seeing more patients with emerging resistance, part of the resistance mechanism enables rewiring of the mitochondrial metabolism pathway. Our data demonstrate that the mitochondrial respiration is reinstated in samples that develop resistance and this mechanism requires further investigation. Future treatments need to keep this in mind and thus we need to consider this adaptation as we design novel therapies. Mitochondrial respiration is not often employed as a preclinical screening tool. We believe this to be an adjunct to traditional viability measurements in preclinical work.

Alternative solutions currently being considered are a shift towards a time limited treatment with venetoclax based regimens to achieve depth of response, decrease time on therapy, and thus reduced costs. These treatment strategies remain resource intensive and not all patients may have easy access in the same manner that ibrutinib was accessible due to the need for admission, transportation to clinics for frequent monitoring, and/or inpatient bed availability. Clinical trials evaluating dose reductions in a randomized fashion are needed and are beginning. Finally, we believe mitochondrial respiration may be a biomarker for disease response to therapy and resistance and thus should be evaluated in the pre-clinical arena for therapeutic development.

## 4. Materials and Methods

CLL patients were divided into two groups: Low and standard dose ibrutinib groups [6]. Low dose was defined as treatment initiated at 140 or 280 mg, and standard dose at 420 mg orally once daily and maintained at those dose levels. Doses were evaluated retrospectively from a clinical cohort and were based on physician preference or toxicity [6]. Protocol requested patients provide samples weekly in the first 4 weeks, and then once monthly. Samples were collected at 1–5 months when it was possible, unless stated otherwise. Patients who had progressed on ibrutinib did not have a matched pre-treatment sample, nor an on treatment mitochondrial profile, as we did not have access to the technology at that time. Clinical patient characteristics and treatments are described in the Supplementary Table S1. The study was approved by the local research ethics committee of the University of Manitoba REB# H2019288. Written informed consent was obtained from all patients. The study was performed in accordance with the Declaration of Helsinki.

Mitochondrial respiration of freshly isolated CLL cells in suspension from patients prior to or on ibrutinib therapy was measured using the high resolution Oroboros oxygraph (Oroboros Instruments GmbH, Innsbruck, Austria) [9,14]. In brief, an Oroboros oxygraph is a Clarke-type oxygen electrode that has two chambers (2 ml volume) equipped with oxygen sensors. Air calibration of these oxygen sensors is performed routinely on any day before starting a respirometric experiment. Freshly isolated lymphocytes were washed once in RPMI 1640 media at 335 Xg for 5 min at room temperature, and then resuspended in the same media. Oxygen consumption rate (OCR) served as a surrogate for mitochondrial electron transport chain function. OCR was measured at baseline and following sequential treatments with the ATP synthase inhibitor oligomycin, uncoupler carbonyl cyanide-p-(trifluoromethoxy) phenylhydrazone (FCCP) to remove the pH gradient and enable maximal rates of electron transport to occur, and antimycin A to block respiratory electron flux at Complex III. After measurement of basal respiration rates, the following chemicals were added: oligomycin (2 µM), FCCP (2–12.5 µM), and antimycin A (2 µM). The mitochondrial respiration parameters are defined as: Baseline respiration is termed basal respiration; maximal respiration is achieved by the addition of the uncoupler FCCP, and spare respiratory capacity is the potential respiratory capacity of the CLL cell to handle stressed conditions and is the difference between the maximal and basal respiration rates. Respiratory control ratio is defined as the ratio of uncoupled respiration and oligomycin-treated respiration rates. Oroboros DatLab software was used to calculate the OCR. Ten million CLL cells were used for all oxygraph experiments.

Plasma CCL3 and CCL4 levels were measured pre and post treatment where matched plasma was available (Meso Scale Diagnostics, Rockville, MD [9,11]. Primary CLL cells from ibrutinib treated patients were lysed and immunoblotted using antibodies targeting pBTK (Tyr223), tBTK, cleaved caspase-3 and uncleaved caspase-3 (Cell Signaling Technology, Danvers, MA), and vinculin (AbCam, Toronto, Canada) served as a loading control. Densitometry is listed in Table S2.

Clinical β-2 M and LDH levels (normal ranges: 1.1–2.4 mg/L and 120–230 U/L, respectively) were obtained to match with the date of sample collection and were used to determine the correlation between the drug effect and level measured in the plasma.

## 5. Conclusions

Mitochondrial respiration in CLL cells parallels disease activity, correlates with chemokines, β-2 M, and LDH at low or standard dose of ibrutinib. In addition, the increase of mitochondrial respiration and a rise in β-2 M and LDH in patients that have progressed on therapy further supports mitochondrial respiration of CLL cells as a biomarker of active disease. We believe mitochondrial respiration can serve as a preclinical tool that can help identify novel compounds in the future, which can be used in parallel with standard available tools.

## Data Availability

Data are contained within the article or supplementary material.

## Supplementary Materials

The following are available online at https://www.mdpi.com/2072-6694/13/2/354/s1, Figure S1: Mitochondrial respiration profiles are time dependent and dose independent of ibrutinib, Figure S2: Increased protein levels of BTK and loss of caspase-3 in ibrutinib-resistant patients, Table S1: CLL patient characteristics, Table S2: Densitometry data of Western blots.

## References

[1] Shanafelt T.D., Wang X.V., Kay N.E., Hanson C.A., O’Brien S., Barrientos J., Jelinek D.F., Braggio E., Leis J.F., Zhang C.C. (2019). Ibrutinib-Rituximab or Chemoimmunotherapy for Chronic Lymphocytic Leukemia. N. Engl. J. Med..

[2] O’Brien S., Jones J.A., Coutre S.E., Mato A.R., Hillmen P., Tam C., Osterborg A., Siddiqi T., Thirman M.J., Furman R.R. (2016). Ibrutinib for patients with relapsed or refractory chronic lymphocytic leukaemia with 17p deletion (RESONATE-17): A phase 2, open-label, multicentre study. Lancet Oncol..

[3] Bose P., Chen L.S., Gandhi V. (2019). Ibrutinib dose and clinical outcome in chronic lymphocytic leukemia—Learning from the ‘real world’. Leuk. Lymphoma.

[4] Bose P., Gandhi V.V., Keating M.J. (2016). Pharmacokinetic and pharmacodynamic evaluation of ibrutinib for the treatment of chronic lymphocytic leukemia: Rationale for lower doses. Expert Opin. Drug Metab. Toxicol..

[5] Chen L.S., Bose P., Cruz N.D., Jiang Y., Wu Q., Thompson P.A., Feng S., Kroll M.H., Qiao W., Huang X. (2018). A pilot study of lower doses of ibrutinib in patients with chronic lymphocytic leukemia. Blood.

[6] Uminski K., Brown K., Bucher O., Hibbert I., Dhaliwal D.H., Johnston J.B., Geirnaert M., Dawe D.E., Banerji V. (2019). Descriptive analysis of dosing and outcomes for patients with ibrutinib-treated relapsed or refractory chronic lymphocytic leukemia in a Canadian centre. Curr. Oncol..

[7] Parikh S.A., Achenbach S.J., Call T.G., Rabe K.G., Ding W., Leis J.F., Kenderian S.S., Chanan-Khan A.A., Koehler A.B., Schwager S.M. (2020). The impact of dose modification and temporary interruption of ibrutinib on outcomes of chronic lymphocytic leukemia patients in routine clinical practice. Cancer Med..

[8] Mato A.R., Timlin C., Ujjani C., Skarbnik A., Howlett C., Banerjee R., Nabhan C., Schuster S.J. (2018). Comparable outcomes in chronic lymphocytic leukaemia (CLL) patients treated with reduced-dose ibrutinib: Results from a multi-centre study. Br. J. Haematol..

[9] Roy Chowdhury S., Bouchard E.D.J., Saleh R., Nugent Z., Peltier C., Mejia E., Hou S., McFall C., Squires M., Hewitt D. (2020). Mitochondrial Respiration Correlates with Prognostic Markers in Chronic Lymphocytic Leukemia and Is Normalized by Ibrutinib Treatment. Cancers.

[10] Ponader S., Chen S.S., Buggy J.J., Balakrishnan K., Gandhi V., Wierda W.G., Keating M.J., O’Brien S., Chiorazzi N., Burger J.A. (2012). The Bruton tyrosine kinase inhibitor PCI-32765 thwarts chronic lymphocytic leukemia cell survival and tissue homing in vitro and in vivo. Blood.

[11] Burger J.A., Quiroga M.P., Hartmann E., Burkle A., Wierda W.G., Keating M.J., Rosenwald A. (2009). High-level expression of the T-cell chemokines CCL3 and CCL4 by chronic lymphocytic leukemia B cells in nurselike cell cocultures and after BCR stimulation. Blood.

[12] Thompson P.A., O’Brien S.M., Xiao L., Wang X., Burger J.A., Jain N., Ferrajoli A., Estrov Z., Keating M.J., Wierda W.G. (2016). beta2 -microglobulin normalization within 6 months of ibrutinib-based treatment is associated with superior progression-free survival in patients with chronic lymphocytic leukemia. Cancer.

[13] Cervantes-Gomez F., Kumar Patel V., Bose P., Keating M.J., Gandhi V. (2016). Decrease in total protein level of Bruton’s tyrosine kinase during ibrutinib therapy in chronic lymphocytic leukemia lymphocytes. Leukemia.

[14] Roy Chowdhury S.K., Smith D.R., Saleh A., Schapansky J., Marquez A., Gomes S., Akude E., Morrow D., Calcutt N.A., Fernyhough P. (2012). Impaired adenosine monophosphate-activated protein kinase signalling in dorsal root ganglia neurons is linked to mitochondrial dysfunction and peripheral neuropathy in diabetes. Brain.

