# Supplementary material for: Ex Vivo Mitochondrial Respiration Parallels Biochemical Response to Ibrutinib in CLL Cells
Source: Cancers (Basel). 2021 Jan 19;13(2):354. doi: 10.3390/cancers13020354 (PMC7835851; doi:10.3390/cancers13020354)
Supplement: Supplementary file 1 [file cancers-13-00354-s001.zip › supplementary data revision/figure S 1 AND 2.pdf]

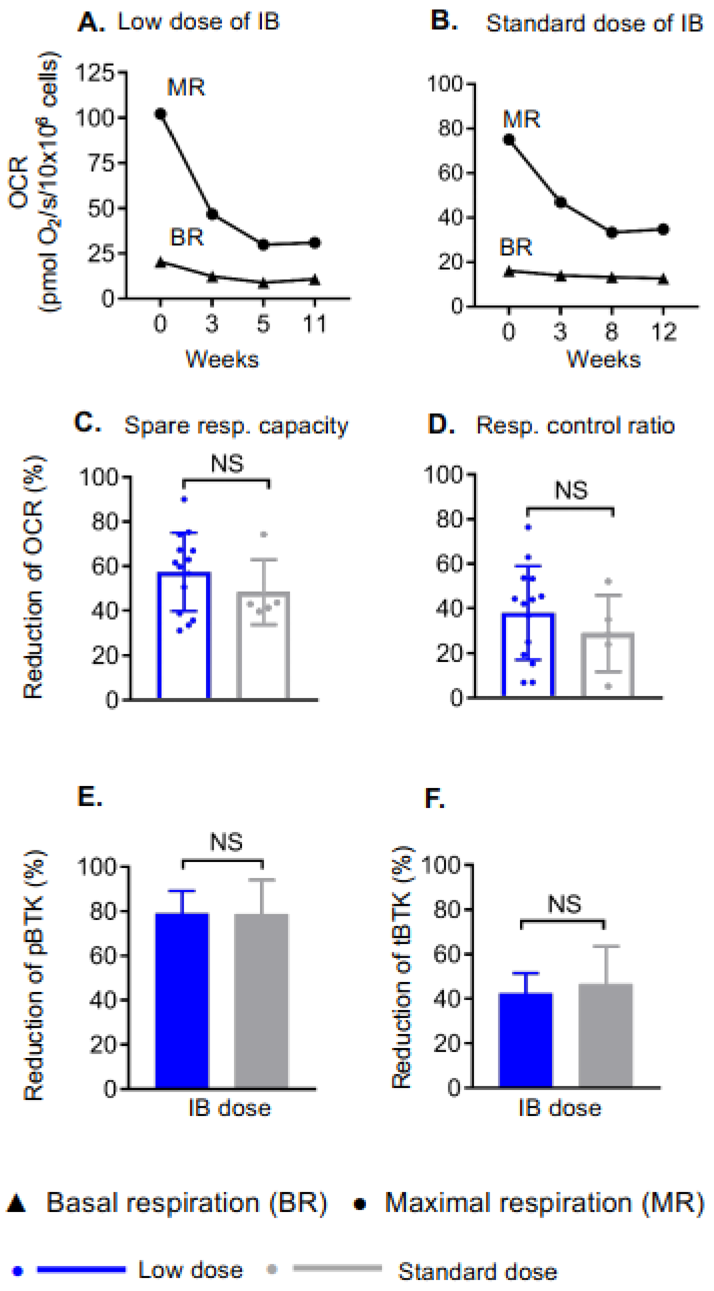

Supplementary Figure S1. Mitochondrial respiration profiles are time dependent and dose independent of ibrutinib. Time-dependent effect of ibrutinib on mitochondrial basal and maximal respiration with low (A) or standard (B) dose. The reduction of maximal (MR) and basal (BR) mitochondrial respiration parameters with low and standard dose of ibrutinib treatment for a sample patient. The effects on spare respiratory capacity (C) and respiratory control ratio (D) in ibrutinib treated samples by dose reveals no difference. N = 14 for low dose and N = 5 for standard dose of ibrutinib. Values are mean ± S.D. (paired two-tailed Student's *t*-test). Similar reduction in pBTK (E) and tBTK (F) levels in samples from low or standard dose ibrutinib treated patients is summarized. N = 8 for low dose and N = 4 for standard dose of ibrutinib. Values are mean ± S.E.M (unpaired two-tailed Student's *t*-test).

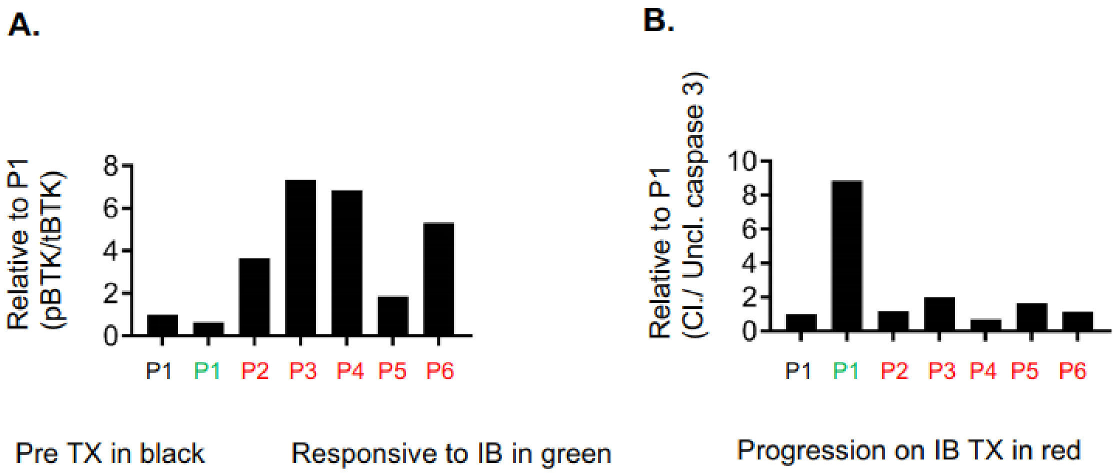

Supplementary Figure S2. Increased protein levels of BTK and loss of caspase-3 in ibrutinib-resistant patients. Quantification of protein levels of pBTK/tBTK ratios (A) and cleaved/uncleaved caspase-3 ratios (B) in ibrutinib-sensitive (P1 in green) and ibrutinib-progressed (P2-P6 in red) compared to pretreatment sample (P1 in black) are shown.
