# Supplementary figures and images for: Ex Vivo Mitochondrial Respiration Parallels Biochemical Response to Ibrutinib in CLL Cells
Source: Cancers (Basel). 2021 Jan 19;13(2):354. doi: 10.3390/cancers13020354 (PMC7835851; doi:10.3390/cancers13020354)

Supplement to Figure 1M

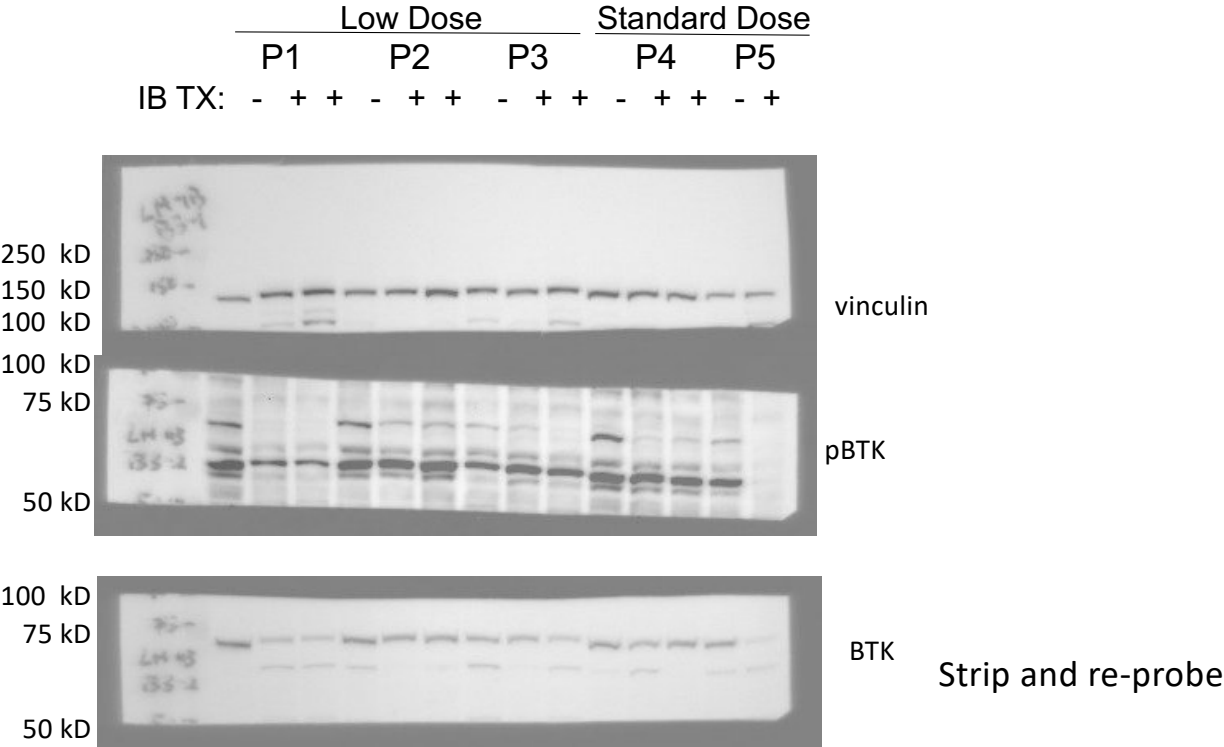

Supplement to Figure 1N

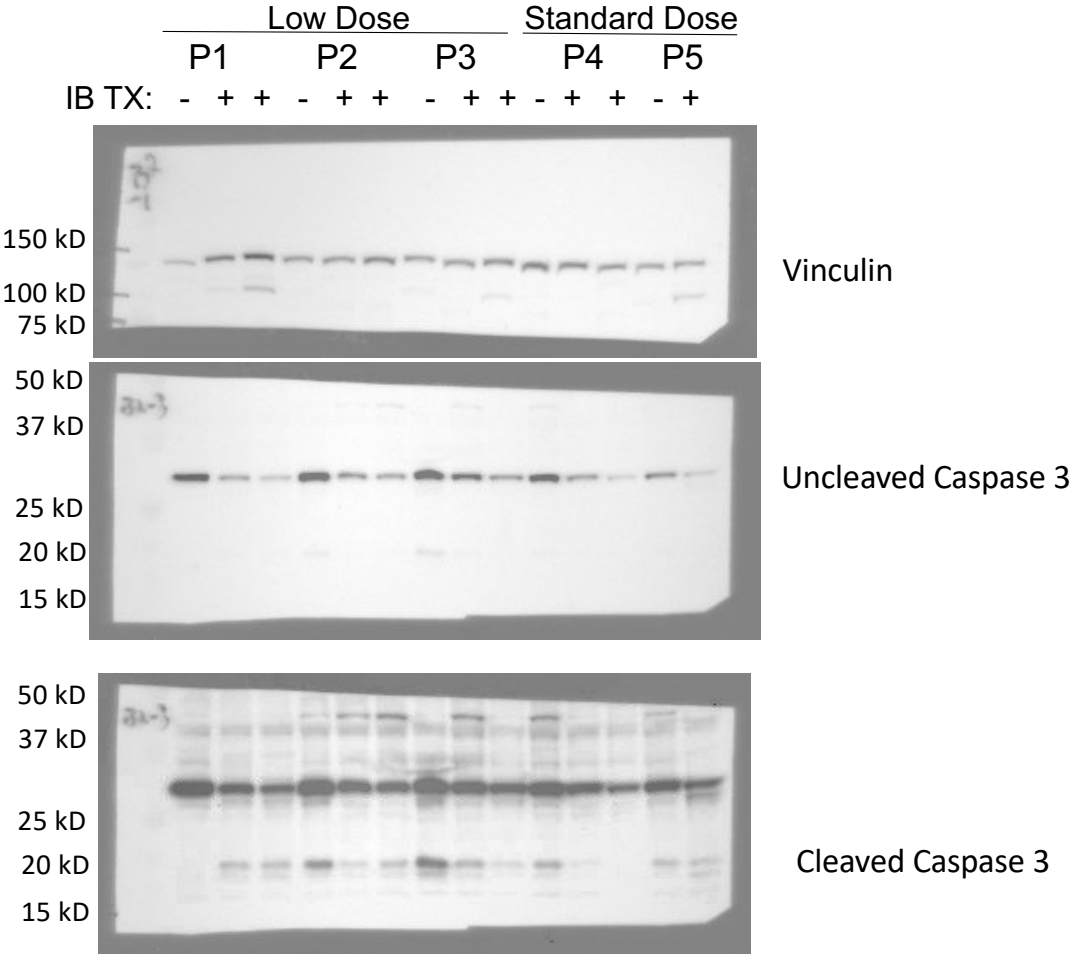

Supplement: Supplementary file 1 [file cancers-13-00354-s001.zip › supplementary data revision/1M pBTK-BTK Fig 1N Caspase cleavage.pdf]

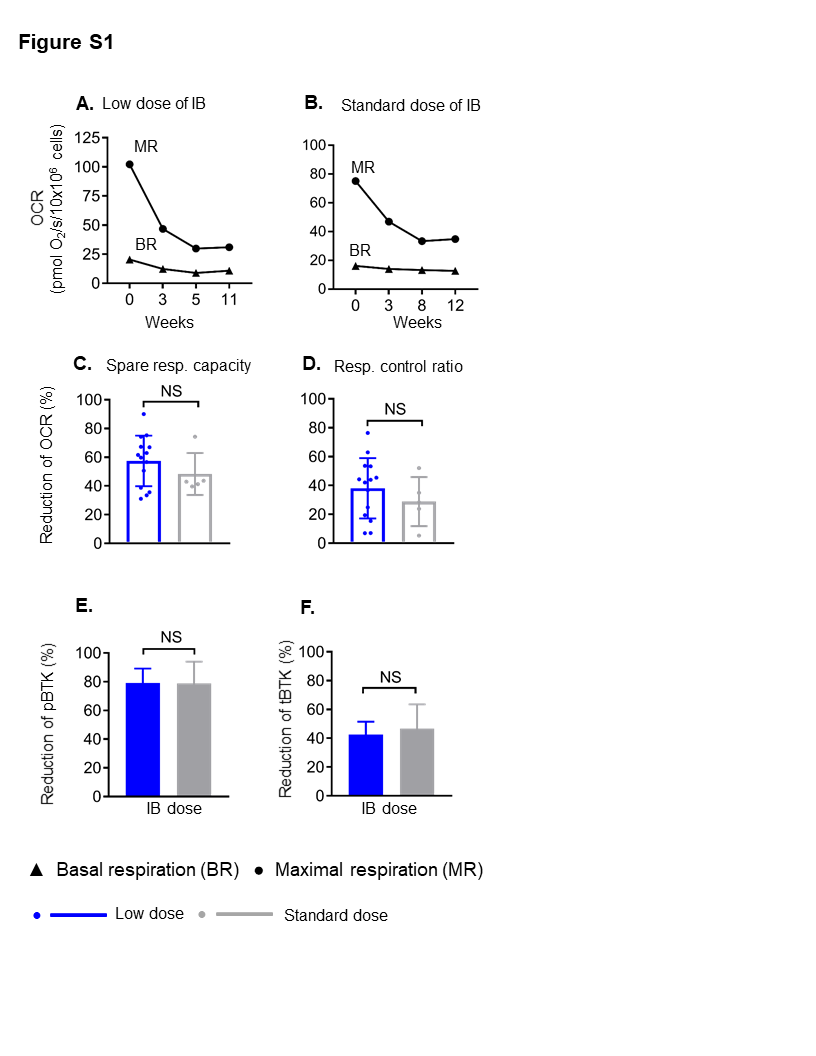

Supplement: Supplementary file 1 [file cancers-13-00354-s001.zip › supplementary data revision/Figure S1.tif]

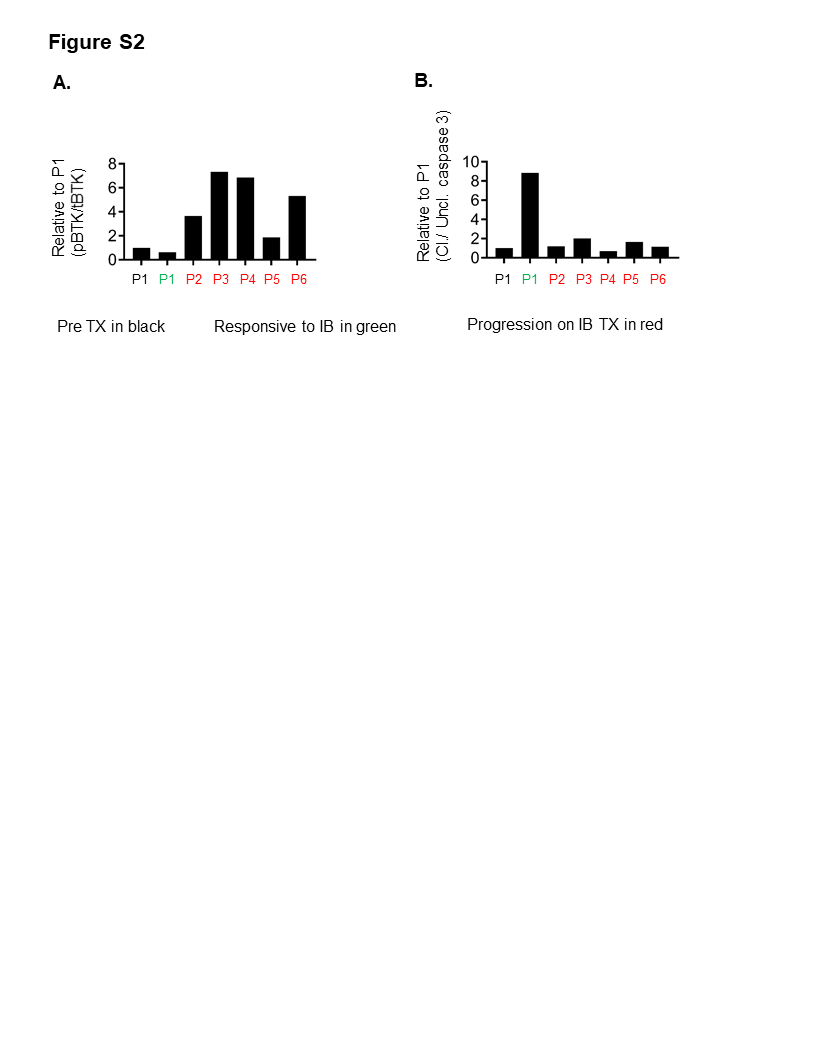

Supplement: Supplementary file 1 [file cancers-13-00354-s001.zip › supplementary data revision/Figure S2.tif]

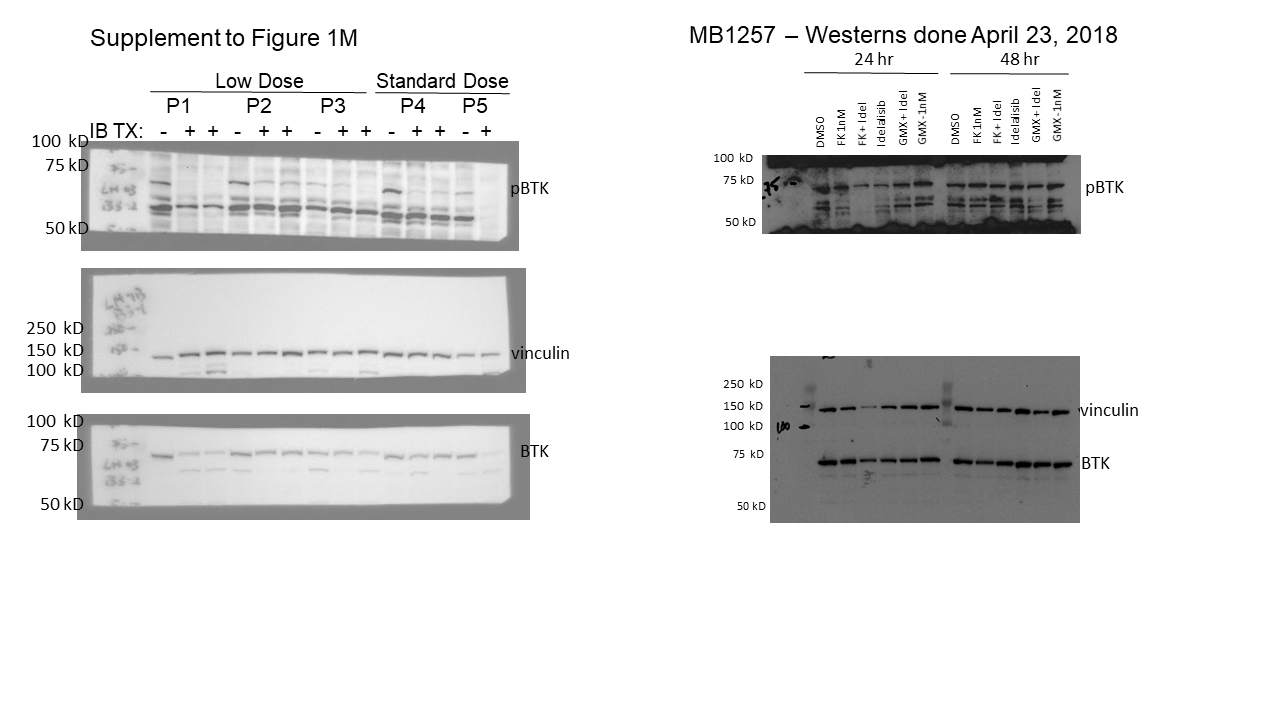

Supplement: Supplementary file 1 [file cancers-13-00354-s001.zip › supplementary data revision/Slide1.TIF]

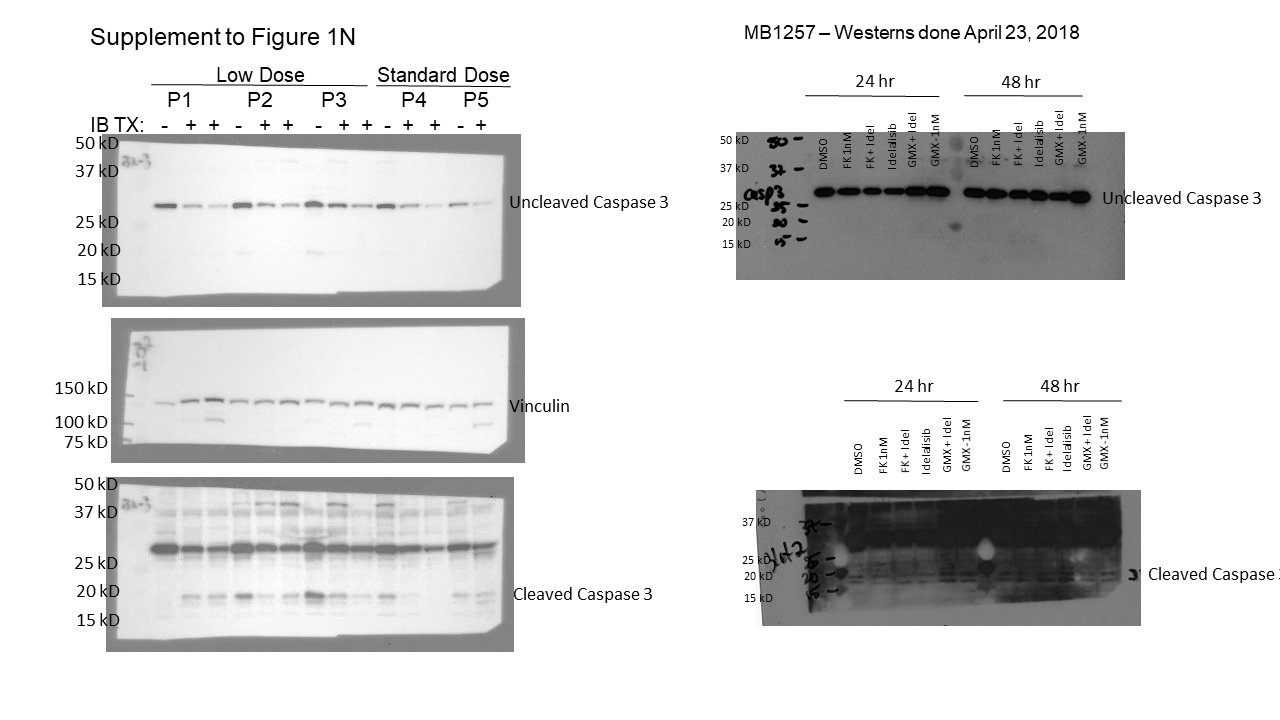

Supplement: Supplementary file 1 [file cancers-13-00354-s001.zip › supplementary data revision/Slide2.TIF]

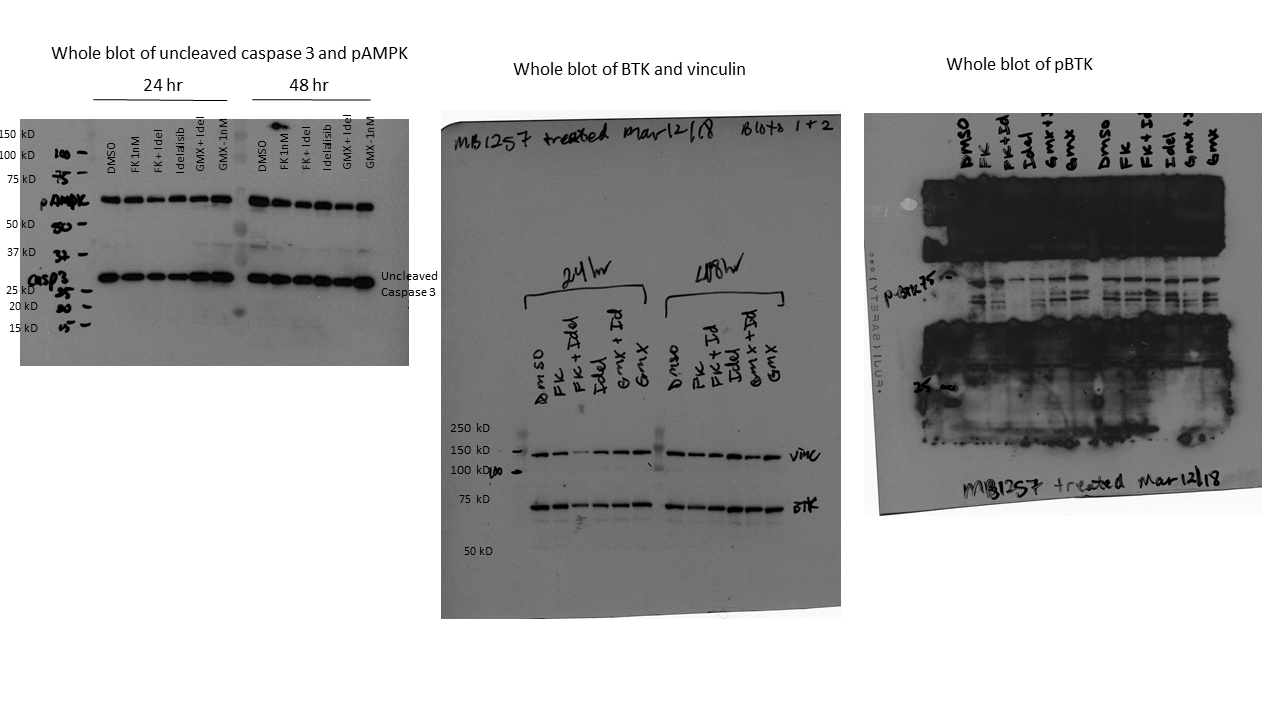

Supplement: Supplementary file 1 [file cancers-13-00354-s001.zip › supplementary data revision/Slide3.TIF]
